# Stable RuIr Nanoalloy Catalyst for Levulinic Acid Hydrogenation Reaction

**DOI:** 10.3390/molecules30010093

**Published:** 2024-12-29

**Authors:** Jingru Wang, Xianshu Dong, Yuping Fan, Yingyong Wang, Xiangyun Guo

**Affiliations:** 1 College of Mining Engineering, Taiyuan University of Technology, Taiyuan 030024, China; dongxianshu@tyut.edu.cn (X.D.); fanyuping@tyut.edu.cn (Y.F.); 2 State Key Laboratory of Coal Conversion, Institute of Coal Chemistry, Chinese Academy of Sciences, Taiyuan 030001, China; wangyy79@sxicc.ac.cn; 3 School of Petrochemical Engineering, Changzhou University, Changzhou 213164, China; xyguo@sxicc.ac.cn

**Keywords:** RuIr alloy, levilinic acid, hydrogenation, γ-valerolactone

## Abstract

Hydrogenation of levulinic acid (LA) represents a significant approach for producing the high-value biomass-based platform compound γ-valerolactone (GVL). In this study, an efficient RuIr alloy bimetallic catalyst supported on SiC was synthesized and applied for the aqueous hydrogenation of LA into GVL under mild conditions. The RuIr/SiC catalyst exhibited high LA conversion and GVL selectivity (both > 99%) in water at 0.2 MPa H_2_ pressure and 25 °C. The excellent performance is attributed to the synergistic interaction between Ru and Ir nanoparticles on the semiconducting SiC support. Furthermore, the catalytic activity of the RuIr/SiC alloy remained basically unchanged after five cycles, confirming the high stability of the bimetallic alloy catalyst.

## 1. Introduction

Primary energy sources, including coal, oil, and natural gas, present challenges to sustainable development due to their non-renewable nature, limited availability, and associated ecological and environmental issues. To promote rational resource utilization and sustainable development, it is urgent to seek environmentally friendly renewable resources and convert waste into valuable materials [1,2,3]. Biomass, as a representative new energy source, is the only renewable green organic carbon source on earth. It serves as a substitute for oil in the production of various fuels, chemicals, and carbon-based materials, drawing considerable attention [4].

**Scheme 1:**
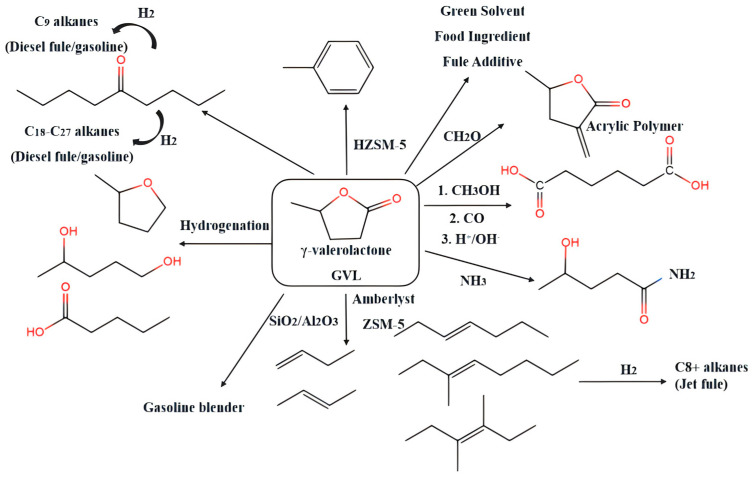
Applications of GVL.

Lignocellulose, the primary component of biomass, can be converted into a variety of platform compounds and subsequently into high-value chemicals through processes such as hydrogenation, deoxygenation, decarbonylation, and so on. Levulinic acid (LA), derived from lignocellulose through multistep hydrolysis, is a key biomass-based platform compound with applications in producing pharmaceuticals, resins, spices, coatings, and other high-value chemicals [5,6,7,8]. γ-Valerolactone (GVL), obtained by hydrogenating LA and its esters, is another crucial biomass platform molecule. GVL is non-toxic and biodegradable, and it finds applications as a solvent and fuel additive, and in the food industry, among others [9,10,11] (Scheme 1).

Ru-based heterogeneous catalysts are widely employed for the hydrogenation of LA to GVL. However, Ru/C catalysts face issues of deactivation due to carbonaceous deposits, leading to reduced stability. Additionally, carbon supports often require multiple regeneration cycles, and Ru nanoparticles on these supports are thermodynamically unstable, tending to aggregate into larger particles under high-temperature conditions [12,13,14,15]. Ir-based catalysts also exhibit good catalytic performance for this reaction [16,17], though their use often necessitates high temperatures or high pressures. Metal nanoalloy catalysts have shown superior catalytic performance due to their unique metal structures and synergistic effects between two or more metals, which have been the focus of research in recent years [15,18,19,20,21,22,23,24]. These studies indicate that metal nanoalloy catalysts exhibit improved activity and stability compared to single-metal catalysts.

Selecting an appropriate support material is critical for catalytic performance. In Ru-based catalysts supported on metal oxide supports (such as Al_2_O_3_, SiO_2_, MCM-41, SBA-15, TiO_2_, and CeO_2_), the exposed hydroxyl groups (–OH) on the oxide surface tend to rehydrate in acidic aqueous environments. This rehydration results in the collapse of the support structure, thereby decreasing catalyst efficiency and potentially causing deactivation [25,26,27]. In contrast, SiC has higher mechanical strength, thermal conductivity, and acid-resistance compared with the classical oxide-based support (e.g., Al_2_O_3_ and SiO_2_), which makes it still able to maintain its original structure to support active components and complete catalytic tasks even in harsh reaction [28,29,30,31]. Consequently, SiC has emerged as a promising support material in heterogeneous catalysis. In our previous work, a highly dispersed Ir/SiC catalyst was prepared and showed excellent catalytic activity and stability for the aqueous hydrogenation of LA to GVL at 50 °C and 0.2 MPa of H_2_ pressure [16].

Building on this foundation, RuIr alloy catalysts supported on high specific surface area SiC were synthesized in this study for LA hydrogenation. The physicochemical properties of the synthesized catalysts and the impact of thermal treatment on catalyst performance were studied. Additionally, the effects of support’s chemical composition were also investigated. The findings indicate that SiC enhances LA hydrogenation activity through its interaction with Ru and Ir metals, while thermal treatment further improves catalyst stability.

## 2. Results and Discussion

### 2.1. Synthesis and Characterization

Table 1 shows the physicochemical properties of RuIr/SiC catalysts treated at different calcination times. SiC has a higher specific surface area (Table 1, entry 1), which decreases after the RuIr loading process. Compared to the Ru_0.5_Ir_2.5_/SiC-0 catalyst directly reduced without calcination, the specific surface area of calcined catalysts is further decreased. Among these, the Ru_0.5_Ir_2.5_/SiC-12 catalyst displays the lowest specific surface area and pore volume (Table 1, entry 6). This reduction can be attributed to the aggregation of RuIr species as calcination time increases, resulting in surface coverage and pore blockage of the SiC support. This observation is corroborated by TEM images (Figure S1).

**Figure 1:**
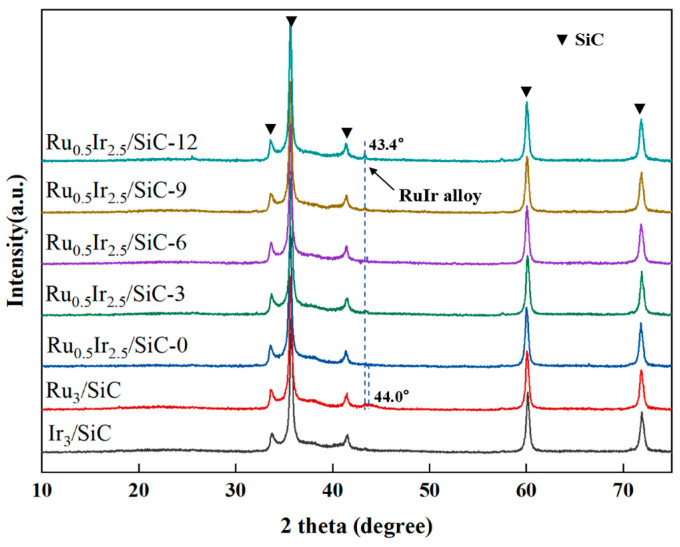
XRD patterns of different RuIr/SiC catalysts.

**Figure 2:**
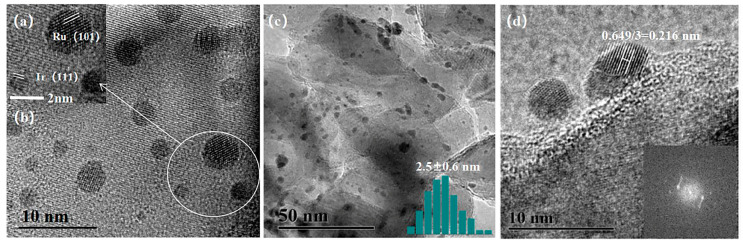
TEM images of Ru_0.5_Ir_2.5_/SiC-0 (**a**,**b**), and Ru_0.5_Ir_2.5_/SiC-6 (**c**,**d**). The inset of (**c**) is corresponding size distribution of RuIr particles.

The XRD patterns of RuIr/SiC catalysts treated at different calcination times are shown in Figure 1. The diffraction peaks at 35.7°, 41.4°, 60.1°, 71.8°, and 75.6° (2θ) correspond to the characteristic peaks of β-SiC, specifically the (101), (111), (200), (220), (311), and (222) planes, respectively [32]. For the Ir_3_/SiC catalyst, no diffraction peaks of Ir are detected, likely due to the highly dispersed and small-sized iridium particles on the SiC surface. In the Ru_3_/SiC catalyst, a weak diffraction peak at 2θ = 44.0° corresponds to the Ru (101) crystal plane, indicating the presence of metallic Ru. The diffraction peaks of Ru and Ir are not observed for the Ru_0.5_Ir_2.5_/SiC-0 catalyst, which may be due to the fact that Ru and Ir are not formed alloys and are independently dispersed on the SiC surface. The absence of Ru peaks may be attributed to its low loading, while the small size of Ir particles could account for the lack of observable peaks. These findings align with the TEM results (Figure 2). For the RuIr/SiC catalyst reduced after calcination, there is a diffraction peak at 43.4°, indicating the formation of RuIr alloy [33].

Figure 2 shows representative TEM images of Ru_0.5_Ir_2.5_/SiC-0 and Ru_0.5_Ir_2.5_/SiC-6 catalysts. The TEM images of Ru_0.5_Ir_2.5_/SiC-0 (Figure 2a,b) reveal that Ru and Ir nanoparticles are independently distributed on the SiC surface, with average diameters of about 2.4 nm and 1.5 nm, respectively (Figure S2). The lattice spacings of Ru and Ir nanoparticles in the Ru_0.5_Ir_2.5_/SiC-0 catalyst are 0.205 nm and 0.216 nm, corresponding to the Ru (101) and Ir (111) crystal planes, respectively [34]. In contrast, the Ru_0.5_Ir_2.5_/SiC-6 catalyst exhibits uniformly dispersed bimetallic RuIr nanoparticles on the SiC surface, with an average diameter of about 2.5 nm (Figure 2c). The high-resolution TEM image of Ru_0.5_Ir_2.5_/SiC-6 shows that the lattice spacing of RuIr alloy nanoparticles is about 0.216 nm, corresponding to the Ir (111) crystal planes (Figure 2d). This observation is likely due to the segregation of Ir on the surface of the alloy particles [35]. These findings, combined with the XRD results, confirm the formation of RuIr alloys.

**Figure 3:**
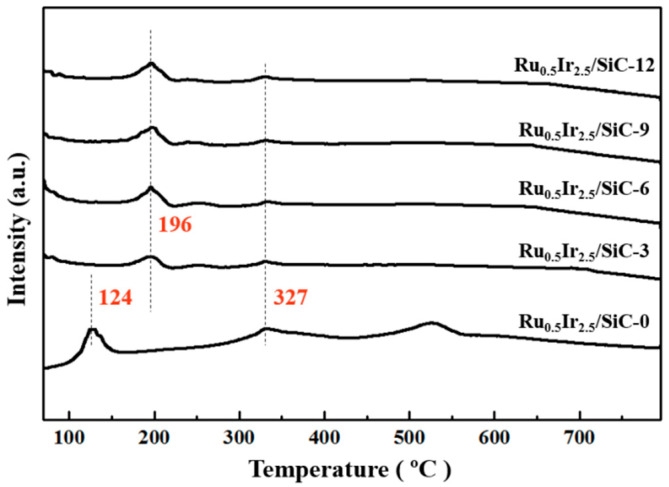
H_2_-TPR profiles of RuIr/SiC catalysts treated with different calcination time.

H_2_-temperature-programmed reduction (H_2_-TPR) was performed to examine the oxidation-reduction properties of the catalysts and the interaction strength between Ru, Ir, and SiC. The H_2_-TPR results (Figure 3) indicate that the reduction peak of the Ru_0.5_Ir_2.5_/SiC-0 catalyst at 124 °C corresponds to the reduction of Ru and Ir species to their metallic forms, Ru^0^ and Ir^0^. The peak observed in the 300–400 °C range is likely associated with the reduction of Ru(OH)_3_ species or RuO_2_, while the peak at about 530 °C is attributed to the strong interaction between ruthenium and the SiC support [36]. After the formation of the RuIr alloy, the reduction temperature in the low-temperature range increases from 124 °C to 196 °C, suggesting an enhanced interaction between the metals and the SiC support. This strengthened interaction facilitates the dispersion and stabilization of RuIr alloy nanoparticles. Additionally, the TPR profiles of the calcined catalysts exhibit a broad feature disappearance at 500–600 °C, which is attributed to the decrease of Ru that strongly interacts with the SiC support after the formation of the RuIr alloy.

### 2.2. Catalytic Performance Studies

The catalytic performance of various catalysts for the hydrogenation of LA into GVL was investigated, and the results are summarized in Table 2. Monometallic Ir_3_/SiC and Ru_3_/SiC catalysts exhibit lower activity, with LA conversions of 61.8% and 55.9%, respectively, at 25 °C (Table 2, entries 1 and 2). When replacing a small amount of Ir by the Ru, the bimetallic Ru_0.5_Ir_2.5_/SiC-0 catalyst demonstrates significantly improved activity (Table 2, entry 3). For the Ru_0.5_Ir_2.5_/SiC-0 catalyst, Ru and Ir nanoparticles are independently distributed on the SiC surface. Due to the electron transferring, electron-rich Ir nanoparticles show stronger ability for H_2_ dissociation, and the SiC surface produces more active sites to accommodate hydrogen species that spill over from Ir and Ru [34]. The Ru_0.5_Ir_2.5_/SiC catalysts treated with different calcination times at 300 °C show varying catalytic activities. After a calcination time of 3 h, the LA conversion decreased to 90.5% (Table 2, entry 4). This may be attributed to the incomplete formation of uniformly dispersed RuIr nanoparticles in the sample, resulting in a decrease in catalytic activity. Extending the calcination time to 6 h, the LA conversion increased to 99.8% (Table 2, entry 5). At this time, RuIr nanoparticles were uniformly dispersed on the SiC surface. However, further prolonging the calcination time reduces the catalytic activity, with LA conversion decreasing to 87.2% and 61.5% for 9 h and 12 h of calcination, respectively (Table 2, entries 6 and 7). This decline in activity may be due to the agglomeration of metal nanoparticles caused by long calcination time.

**Figure 4:**
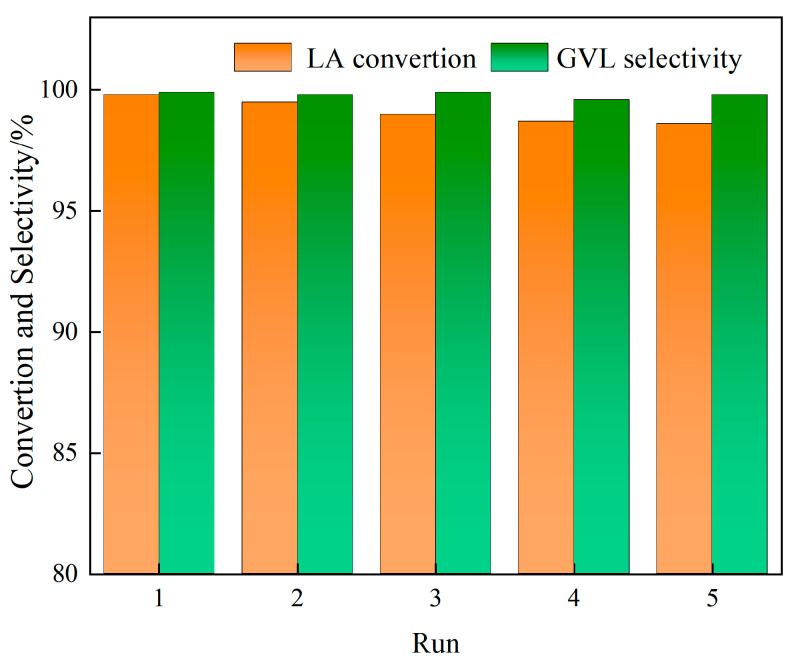
Recyclability in the hydrogenation of LA over the Ru_0.5_Ir_2.5_/SiC-6 catalyst.

Catalyst stability is a crucial factor in evaluating the performance of heterogeneous catalysts. As an acidic substance, LA can corrode catalysts and lead to the loss of active components during reactions, causing deactivation. Thus, enhancing catalyst stability has remained a key research focus for this reaction. The stability of Ru_0.5_Ir_2.5_/SiC catalysts was investigated further in LA hydrogenation. Although Ru_0.5_Ir_2.5_/SiC-0 exhibited excellent initial catalytic activity, its stability was relatively poor. After five cycles, the catalytic activity decreased from 99.9% to 87.0% (Figure S3), which is mainly caused by the agglomeration of active metal particles on the support surface, as observed in the TEM images of the used catalyst (Figure S4). In contrast, the Ru_0.5_Ir_2.5_/SiC-6 catalyst demonstrated high catalytic stability, maintaining nearly constant activity over five consecutive cycles (Figure 4). TEM analysis of the Ru_0.5_Ir_2.5_/SiC-6 catalyst after five reaction cycles (Figure S5) revealed no significant morphological changes or particle agglomeration. Overall, the RuIr/SiC alloy catalyst showed reliable stability in biomass-derived LA hydrogenation. Therefore, the Ru_0.5_Ir_2.5_/SiC-6 catalyst was selected for further studies due to its superior combination of activity and stability.

### 2.3. RuIr/SiC Alloy Nanoparticles for Catalysis

**Figure 5:**
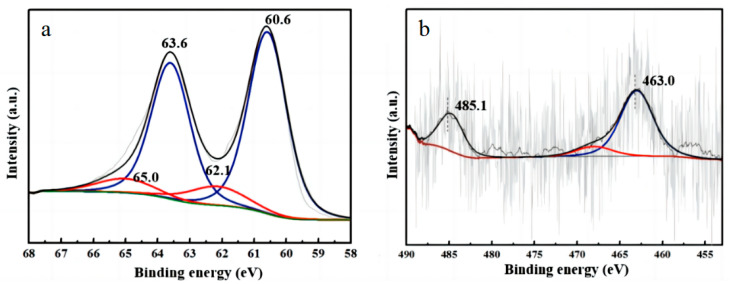
XPS of Ir 4f in Ru_0.5_Ir_2.5_/SiC-6 (**a**) and Ru 3p in Ru_0.5_Ir_2.5_/SiC-6 (**b**).

XPS was conducted to analyze the electronic properties of Ir and Ru in the Ru_0.5_Ir_2.5_/_SiC_-6 catalyst. The XPS results (Figure 5a) show binding energy (BE) peaks at 60.6 eV and 63.6 eV, corresponding to Ir(0) 4f_7/2_ and Ir(0) 4f_5/2_ electronic states, respectively. These values are lower than the standard BE values for metallic Ir (60.9 eV for Ir 4f_7/2_ and 63.9 eV for Ir 4f_5/2_), indicating that Ir particles are electron-enriched. Additionally, BE peaks at 62.1 eV and 65.0 eV correspond to Ir^n+^ 4f_7/2_ and 4f_5/2_ states, indicating partial oxidation of a small amount of Ir in the catalyst. Both Ir^0^ and Ir^n+^ peaks in Ru_0.5_Ir_2.5_/SiC-6 are shifted to lower BE values compared to those of Ir in Ir_3_/SiC (Figure S6a). In Figure 5b, the BE values at 463.0 eV and 485.1 eV correspond to Ru^0^ 3p_3/2_ and Ru^0^ 3p_1/2_ electronic states, respectively. These BE values for Ru in Ru_0.5_Ir_2.5_/SiC-6 are shifted to higher energies compared to Ru in Ru_3_/SiC (Figure S6b). Previous research confirmed that electron transfer occurs from the conduction band of SiC to Ir due to the Mott–Schottky effect in Ir_3_/SiC [16]. In Ru_0.5_Ir_2.5_/SiC-6, this electron enrichment on Ir is further enhanced, suggesting that electron transfer is driven by both the Mott–Schottky contact and electron injection from Ru. This interplay between Ru and Ir contributes to the enhanced electronic properties of the bimetallic catalyst.

**Figure 6:**
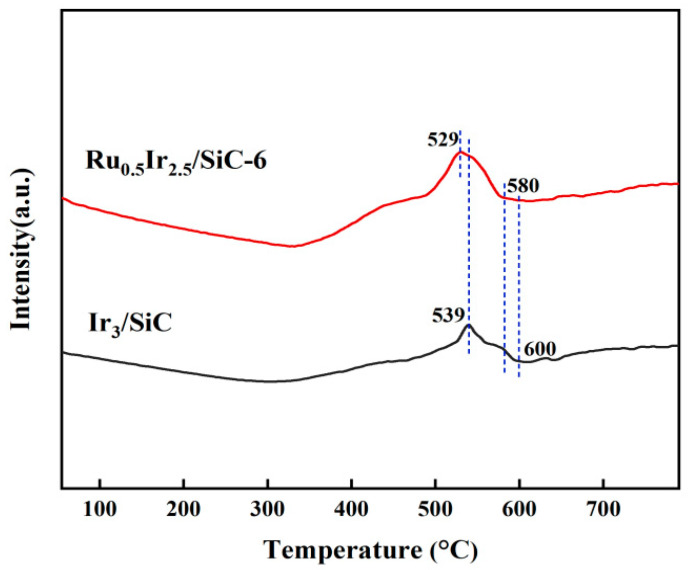
H_2_-TPD profiles of Ir_3_/SiC and Ru_0.5_Ir_2.5_/SiC-6.

H_2_-temperature-programmed desorption (H_2_-TPD) was performed to investigate the strength of hydrogen–surface interactions and the number of active sites for H_2_ adsorption on the catalyst. The H_2_-TPD spectra (Figure 6) for Ir_3_/SiC show a broad H_2_ desorption peak in the range of 320–590 °C, with a maximum at 539 °C, attributed to hydrogen desorption from Ir particles. Additionally, a secondary desorption starting at 600 °C and extending above 800 °C corresponds to direct H_2_ desorption from the SiC surface. These results indicate the presence of at least two types of H_2_ chemisorption sites on the catalyst surface: weak and strong chemisorption sites. For the Ru_0.5_Ir_2.5_/SiC-6 catalyst, the H_2_ desorption temperatures from both sites decrease to 529 °C and 580 °C, respectively. This reduction in desorption temperatures implies weaker interactions between adsorbed hydrogen and the catalyst surface, which facilitates the hydrogenation processes occurring on the catalyst surface. The TPD results demonstrate that RuIr alloy nanoparticles increase the number of active sites for H_2_ chemisorption while weakening the interaction between hydrogen species and the SiC surface, as well as the Ir surface. There are many studies that have shown that hydrogen atoms can be chemisorbed on the SiC surface, and that the migration of hydrogen atoms on SiC has a very low energy barrier [31,37]. In this study, H_2_ dissociates on the Ir surface and subsequently spills over onto the SiC surface. These spillover hydrogen species have a lower migration barrier on the SiC surface, and thus have high activity in chemical reactions.

**Figure 7:**
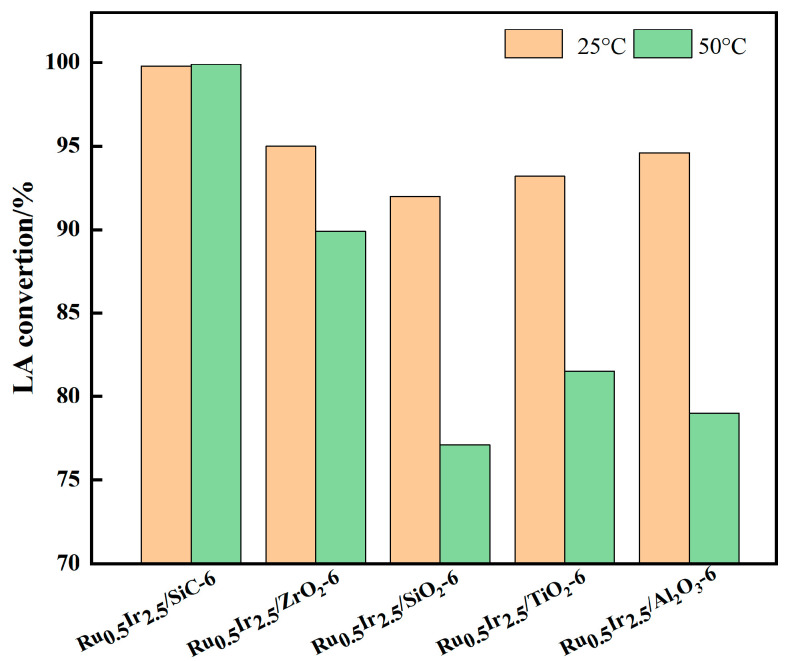
Catalytic performance comparisons of RuIr catalysts with different supports for LA hydrogenation.

The choice of support significantly influences the activity and stability of catalysts, as the interaction between the metal and support enhances catalytic performance. Commonly used supports in catalytic research include metal oxides (e.g., Al_2_O_3_, SiO_2_, TiO_2_, ZrO_2_) and carbon-based materials (e.g., carbon nanotubes, graphene). However, at elevated reaction temperatures, these supports may experience varying levels of corrosion in acidic environments, leading to the detachment of active components from the support. This detachment is a major factor contributing to the reduction in catalyst activity. The catalytic performance of RuIr alloy catalysts with different supports for LA hydrogenation was investigated, and the results are shown in Figure 7. Among the tested catalysts, the Ru_0.5_Ir_2.5_/SiC-6 catalyst exhibited the highest catalytic activity for LA hydrogenation at 25 °C in water under 0.2 MPa H_2_ pressure for 2 h (4 mmol LA). The results indicate that the SiC support enhances the hydrogenation performance of the catalyst. Furthermore, at a reaction temperature of 50 °C (10 mmol LA), SiC demonstrated superior acid resistance, maintaining its structural integrity and catalytic efficiency under acidic conditions.

## 3. Materials and Methods

### 3.1. Materials and Preparation Methods

Iridium chloride hydrate (IrCl_3_·3H_2_O), ruthenium(III) chloride hydrate (RuCl_3_·3H_2_O), LA, ethanol, TiO_2_, ZrO_2_, SiO_2_, and Al_2_O_3_ were procured from Aladdin, while graphene and activated carbon were obtained from TM (Shanxi, Taiyuan, China). Deionized water was prepared in the laboratory. High-purity nitrogen, argon, and hydrogen gas (99%) were supplied by TISCO (Shanxi, Taiyuan, China). All chemicals and reagents were used as received, without further purification.

Bimetallic RuIr/SiC alloy catalysts were synthesized using a straightforward and adaptable co-impregnation method. Cubic SiC powders with a specific surface area of 46 m^2^/g were prepared via a sol-gel and carbothermal reduction process [38]. In a typical synthesis, the SiC support was added to 10 mL of ethanol containing the required quantities of IrCl_3_ (2 mg/mL) and RuCl_3_ (10 mg/mL) solutions and stirred for 4 h at room temperature. The mixture was heated to 80 °C until the solvent evaporated completely. The obtained solid was dried in an oven at 100 °C for 12 h and subsequently calcined at 300 °C at a heating rate of 10 °C/min for 3 h, 6 h, 9 h, and 12 h, respectively. After calcination, the samples were reduced in a 5% H_2_/Ar mixture at 300 °C with a rate of 5 °C/min for 2 h to produce SiC-supported catalysts containing 0.5 wt% Ru and 2.5 wt% Ir. These were labeled as Ru_0.5_Ir_2.5_/SiC-3, Ru_0.5_Ir_2.5_/SiC-6, Ru_0.5_Ir_2.5_/SiC-9, and Ru_0.5_Ir_2.5_/SiC-12, respectively. The catalyst reduced directly without calcination was labeled as Ru_0.5_Ir_2.5_/SiC-0. For comparison, single-metal catalysts (Ru_3_/SiC, Ir_3_/SiC) with a metal content of 3 wt% were synthesized using a similar method.

Catalysts such as Ru_0.5_Ir_2.5_/graphene, Ru_0.5_Ir_2.5_/TiO_2_, Ru_0.5_Ir_2.5_/ZrO_2_, Ru_0.5_Ir_2.5_/SiO_2_, and Ru_0.5_Ir_2.5_/Al_2_O_3_ were prepared following a similar procedure.

### 3.2. Catalysts Characterization

The micromorphology and structure of the catalysts were characterized using JEM-2100F high-resolution transmission electron microscopy (TEM) (JEOL, Tokyo, Japan) at an accelerating voltage of 200 kV. The phase composition and crystal structures were analyzed using X-ray diffraction (XRD) with a Rigaku D-max/RB-2500 diffractometer (Rigaku, Tokyo, Japan), employing CuK_α_ radiation (λ = 1.5406 Å) and a scanning speed of 4°/min. X-ray photoelectron spectroscopy (XPS) were conducted on an ESCALAB 3MKII spectrometer (Shimadzu/Kratos, Milton Keynes, UK) equipped with an Mg K_α_ X-ray source, with peak calibration against the characteristic C 1s peak at 284.8 eV. Nitrogen adsorption–desorption isotherms and pore size distribution curves at 77 K were measured using a TriStar II 3020 instrument. The BET specific surface area (m^2^/g) was determined from nitrogen adsorption–desorption isotherms, and pore size distributions were calculated using the Barrett–Joyner–Halenda (BJH) method.

Hydrogen temperature-programmed reduction (H_2_-TPR) and hydrogen temperature-programmed desorption (H_2_-TPD) were performed using a TP-5080 chemisorption instrument (Tianjin Xianquan, Tianjin, China). The catalysts had particle sizes of 20–40 μm. For H_2_-TPR, 50 mg of the sample was loaded into a quartz tube, and a 10 vol% H_2_/N_2_ mixture was introduced as the reducing gas. The sample was first purged at 30 °C until baseline stabilization, followed by heating at a rate of 10 °C/min for reduction. For H_2_-TPD, 100 mg of catalyst was pretreated in N_2_ flow (25 mL/min) at 300 °C for 2 h, then cooled to room temperature. After pretreatment, H_2_ was introduced to the sample via pulse injection for 10–20 min until adsorption saturation. The system was purged with N_2_ (25 mL/min) until the signal stabilized, followed by linear heating from 30 to 800 °C at a rate of 10 °C/min, with signals detected using TCD.

### 3.3. Catalytic Reaction

The catalytic hydrogenation of LA to GVL was carried out in a 50 mL stainless-steel autoclave equipped with a pressure gauge and automatic temperature control. The typical procedure was as follows: 4 mmol of LA, 50 mg of catalyst, and 10 mL of solvent were added to the autoclave. The reactor was purged with hydrogen three times to remove air, then pressurized with 0.2 MPa H_2_ and maintained at room temperature (25 °C) for 2 h. After the reaction, the reaction mixture was filtered through an organic filtration membrane, and the filtrate was analyzed using gas chromatography-mass spectrometry (GC-MS, BRUKER SCION SQ 456, Germany).

The conversion of LA and the selectivity for GVL were determined using the external standard method, calculated according to Equations (1) and (2) as follows:

(1) $$Conv.(\%)=\frac{M_{0,LA}-M_{t,LA}}{M_{0,LA}}\times 100\%$$

(2) $$Select.(\%)=\frac{M_{t,GVL}}{M_{0,LA}}\times 100\%$$

## 4. Conclusions

In conclusion, a SiC-supported RuIr alloy nanocatalyst was successfully synthesized and characterized using TEM and XPS analyses, revealing uniform dispersion of RuIr alloy nanoparticles on the SiC surface. The RuIr nanoparticles demonstrated efficient catalytic activity for the hydrogenation of LA to GVL under mild conditions, with the exceptional performance attributed to the electronic synergistic effects among Ru, Ir, and SiC. Molecular hydrogen dissociates on the electron-enriched Ir surface, followed by hydrogen spillover onto the SiC surface, thereby enhancing catalytic activity. The catalyst exhibited high stability, maintaining almost consistent catalytic activity and product yield over five consecutive cycles. Compared to RuIr catalysts supported on other supports, the SiC support demonstrated superior acid resistance during the reaction. This study offers a promising approach to biomass conversion, potentially contributing to advancements in industrial production.

## Figures and Tables

**Table 1:** Physicochemical properties of catalysts.

Entry	Catalysts	Calcination Times	*S*_BET_ (m^2^/g)	*V*_P_ (cm^3^/g)	*d*_p_ (nm)
1	SiC	—	45.6	0.13	11.8
2	Ru_0.5_Ir_2.5_/SiC-0	0 h	37.2	0.11	11.8
3	Ru_0.5_Ir_2.5_/SiC-3	3 h	36.8	0.10	11.4
4	Ru_0.5_Ir_2.5_/SiC-6	6 h	36.2	0.11	12.0
5	Ru_0.5_Ir_2.5_/SiC-9	9 h	36.2	0.11	12.6
6	Ru_0.5_Ir_2.5_/SiC-12	12 h	34.5	0.09	10.9

**Table 2:** The catalytic performances of catalysts.

Entry	Catalyst	Conv. (%)	Select. (%)
1	Ir_3_/SiC	61.8	99.8
2	Ru_3_/SiC	55.9	99.8
3	Ru_0.5_Ir_2.5_/SiC-0	99.9	99.9
4	Ru_0.5_Ir_2.5_/SiC-3	90.5	99.7
5	Ru_0.5_Ir_2.5_/SiC-6	99.8	99.8
6	Ru_0.5_Ir_2.5_/SiC-9	87.2	99.9
7	Ru_0.5_Ir_2.5_/SiC-12	61.5	99.6

Reaction conditions: H_2_O (10 mL), levulinic acid (4 mmol), catalyst (50 mg), H_2_ (0.2 MPa), 25 °C, 2 h.

## Data Availability

The authors confirm that the data supporting the findings of this study are available within the article and its supplementary materials.

## Supplementary Materials

The following supporting information can be downloaded at https://www.mdpi.com/article/10.3390/molecules30010093/s1: Figure S1: TEM image of Ru0.5Ir2.5/SiC-12; Figure S2: Size distributions of (a) Ru and (b) Ir nanoparticles in the Ru0.5-Ir2.5/SiC-0 catalyst; Figure S3: Recyclability data for the hydrogenation of LA over the Ru0.5Ir2.5/SiC-0 catalyst; Figure S4: TEM image of the used Ru0.5Ir2.5/SiC-0 catalyst after five reaction cycles; Figure S5: TEM image of the Ru0.5Ir2.5/SiC-6 catalyst after five reaction cycles; Figure S6: XPS analysis of (a) Ir_3_/SiC and (b) Ru_3_/SiC.
