# Supplementary material for: Stable RuIr Nanoalloy Catalyst for Levulinic Acid Hydrogenation Reaction
Source: Molecules. 2024 Dec 29;30(1):93. doi: 10.3390/molecules30010093 (PMC11721684; doi:10.3390/molecules30010093)
Supplement: Supplementary file 1 [file molecules-30-00093-s001.zip › molecules-3309584-supplementary.pdf]

Highly active RuIr/SiC alloy catalyst for aqueous hydrogenation of levulinic acid to γ-valerolactone

Jingru Wang* [a], Xianshu Dong[a], Yuping Fan[a], Yingyong Wang[b], Xiangyun Guo[c]

[a]*College of Mining Engineering, Taiyuan University of Technology, Taiyuan 030024, PR China.*

[b]*State Key Laboratory of Coal Conversion, Institute of Coal Chemistry, Chinese Academy of Sciences, Taiyuan 030001, PR China*

[c]*School of Petrochemical Engineering, Changzhou University, Changzhou 213164, PR China*

Supporting Information

Supporting Information

Figure Captions

Figure S1. TEM image of $Ru_{0.5}Ir_{2.5}$/SiC-12

Figure S2. Size distributions of (a) Ru and (b) Ir nanoparticles in the $Ru_{0.5}$-$Ir_{2.5}$/SiC-0 catalyst.

Figure S3. Recyclability data for the hydrogenation of LA over the $Ru_{0.5}Ir_{2.5}$/SiC-0 catalyst.

Figure S4. TEM image of the used $Ru_{0.5}Ir_{2.5}$/SiC-0 catalyst after five reaction cycles.

Figure S5. TEM image of the $Ru_{0.5}Ir_{2.5}$/SiC-6 catalyst after five reaction cycles.

Figure S6. XPS analysis of (a) Ir_3/SiC and (b) Ru_3/SiC

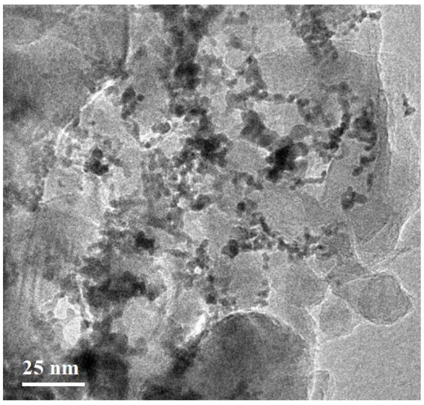

Figure S1. TEM image of $Ru_{0.5}Ir_{2.5}$/SiC-12.

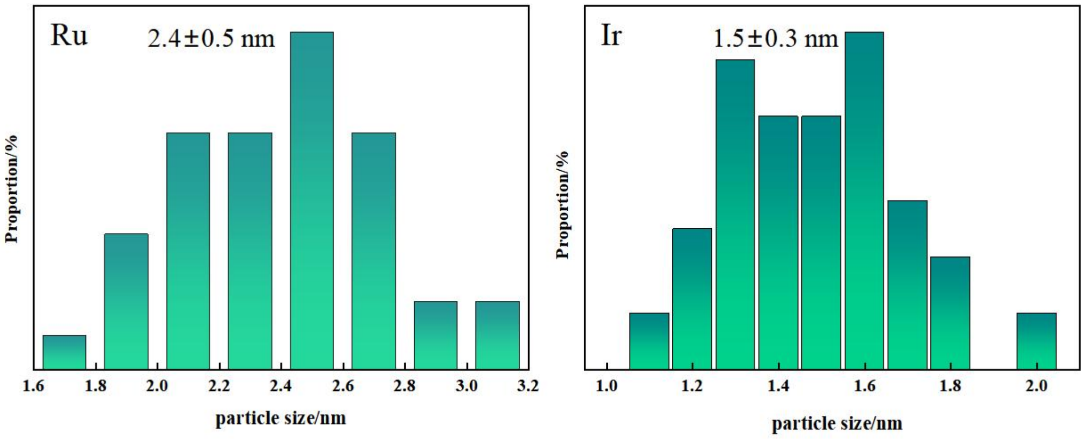

Figure S2. Size distributions of (a) Ru and (b) Ir nanoparticles in the $Ru_{0.5}$-$Ir_{2.5}$/SiC-0 catalyst.

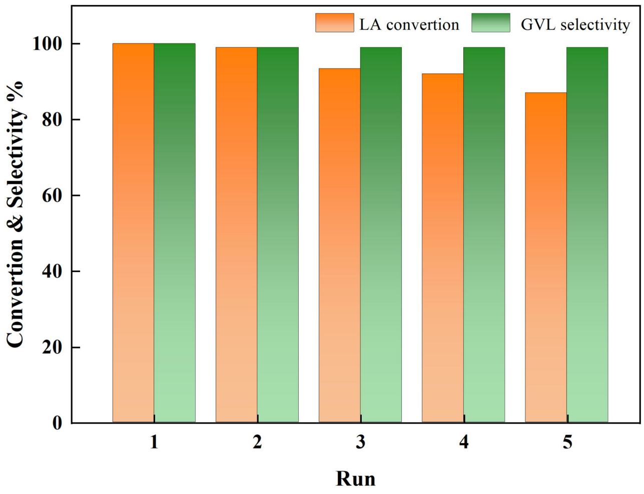

Figure S3. Recyclability data for the hydrogenation of LA over the $Ru_{0.5}Ir_{2.5}$/SiC-0 catalyst.

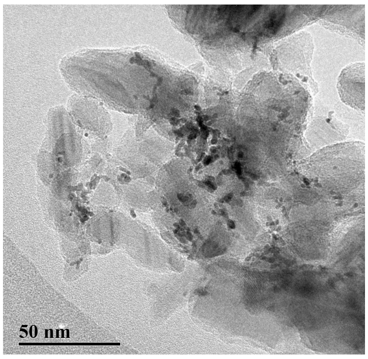

Figure S4. TEM image of the used $Ru_{0.5}Ir_{2.5}$/SiC-0 catalyst after five reaction cycles.

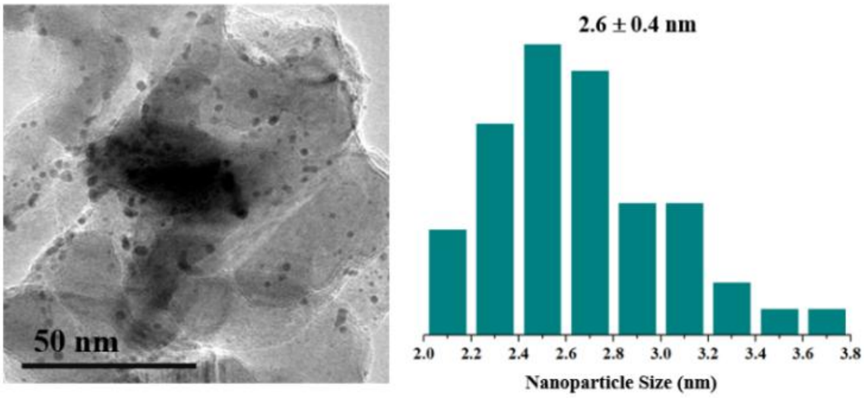

Figure S5. TEM image of the $Ru_{0.5}Ir_{2.5}$/SiC-6 catalyst after five reaction cycles.

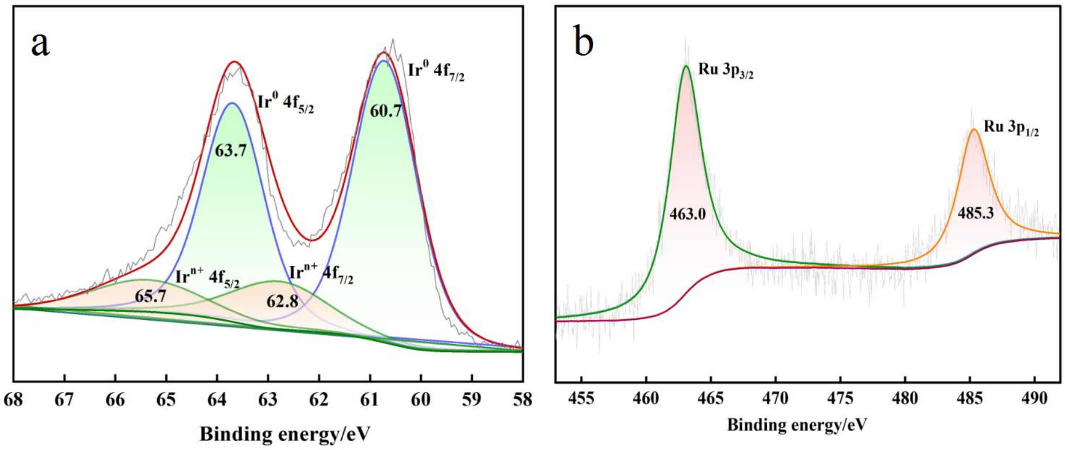

Figure S6. XPS analysis of (a) Ir_3/SiC and (b) Ru_3/SiC
